# A Comprehensive Prospective Analysis of Surgical Outcomes and Adverse Events in Spinal Procedures Among Octogenarians: A Detailed Analysis From a German Tertiary Center

**DOI:** 10.1177/21925682241250328

**Published:** 2024-04-28

**Authors:** Pavlina Lenga, Philip Dao Trong, Vassilios Papakonstantinou, Andreas W. Unterberg, Basem Ishak

**Affiliations:** 1 Department of Neurosurgery, 214802 Heidelberg University Hospital, Heidelberg, Germany

**Keywords:** adverse events, octogenarians, spinal surgery, tertiary care, wound infection

## Abstract

**Study design:**

Prospective case series.

**Objectives:**

Drawing from prospective data, this study delves into the frequency and nature of adverse events (AEs) following spinal surgery specifically in octogenarians, shedding light on the challenges and implications of treating this specific cohort as well as on risk factors for their occurrence.

**Methods:**

Octogenarians who received spinal surgery and were discharged between January 2019 and December 2022 were proactively included in our study. An AE was characterized as any incident transpiring within the initial 30 days after surgery that led to an unfavorable outcome.

**Results:**

From January 2020 to December 2022, 184 octogenarian patients (average age: 83.1 ± 2.8 years) underwent spinal surgeries. Of these, 81.5% were elective and 18.5% were emergencies, with 69.0% addressing degenerative pathologies. Using the Charlson Comorbidity Index, the mean score was 8.1 ± 2.2, highlighting cardiac diseases as predominant. Surgical details show 71.2% had decompression, with 28.8% receiving instrumentation. AEs included wound infections 3.1% for degenerative, 13.3% for tumor and dural leaks. The overall incidence of dural leaks was found to be 2.7% (5/184 cases), and each case underwent surgical revision. Pulmonary embolism resulted in two fatalities post-trauma. Wound infections (26.7%) were prevalent in infected spine cases. Significant AE risk factors were comorbidities, extended surgery durations, and instrumentation procedures.

**Conclusions:**

In octogenarian spinal surgeries, AEs occurred in 15.8% of cases, influenced by comorbidities and surgical complexities. The 2.2% mortality rate wasn’t linked to surgeries. Accurate documentation remains crucial for assessing outcomes in this age group.

## Introduction

With the global increase in life expectancy due to advancements in health care and enhanced quality of life, the proportion of the world’s population aged over 60 is projected to rise sharply, from 12% to 22%.^1^ One of the salient implications of this demographic shift is the pronounced susceptibility of older individuals, especially those post-surgery, to elevated rates of morbidity and mortality.^2-4^ The decision to operate on such patients remains contentious, compounded by the absence of clear guidelines for spinal diseases based on patient age. Consequently, there is often a reliance on institutional standards and individual surgeons’ expertise in formulating therapeutic concepts. Previous studies have indicated favorable outcomes for older patients with conditions like degenerative lumbar diseases, albeit within certain cohorts.^5-9^ Given the amplified risks inherent to this population, complications can lead to increased economic burdens, heightened disability, and in severe cases, fatality.^10,11^ Post-operative complications in the elderly have been documented to vary between 3% and 29%, contingent on the pathology and surgical technique.^12-19^ This underscores the pressing necessity to elucidate complications and their etiological mechanisms to significantly curtail morbidity and mortality rates. Recent developments highlight the emergence of morbidity and mortality conferences (MMC) as a mechanism to preempt or mitigate recurrent complications.^20^ Notably, the majority of these investigations rely predominantly on retrospective data which compromises their validity. Furthermore, our findings suggest that the relative rarity of adverse events in spinal surgery may be attributed to the rigorous documentation of such occurrences, which enhances the continual refinement of safety protocols and the proactive implementation of preventative measures.^21^ However, a palpable gap exists: the lack of comprehensive, prospective data centered exclusively on older patients, especially octogenarians. Filling this void is paramount, not only to establish rigorous care guidelines but also to optimize resource and funding allocation, foster interdisciplinary collaborations, and ultimately diminish the frequency of AEs.

To address this existing gap, we have initiated the creation of a prospectively assembled database from a premier neurological tertiary center, emphasizing spine interventions in octogenarians. Therefore, our aim is to evaluate their clinical trajectory, morbidity and mortality rates, and identify potential risk factors for the occurrence of AEs

## Methods

In this study, we adopted a prospective approach, sourcing data from a single-center, neurosurgical tertiary care facility. The study was conducted under the approval of our institution’s ethics committee (reference S-425/2022) and aligned with the ethical guidelines set by the Declaration of Helsinki. In this specific context, there was no requirement for obtaining individual informed consents as the completion of POPAE forms is an inherent aspect of our standard institutional protocol. As such, it seamlessly merges with our facility’s routine operations, negating the need for additional permissions. We adhered to data collection and analysis procedures as outlined in our earlier publications.^3,21^ This method entailed an in-depth comparative evaluation between our forward-looking Post-Operative Adverse Event (POPAE) database and the broader administrative hospital records. Moreover, our regular quarterly assessments serve as a check mechanism, enabling the identification and rectification of any statistical deviations, bolstering our data’s credibility. As delineated by our team in prior works, our database is persistently maintained and updated by 15 board-certified neurosurgeons and 18 neurosurgical residents. Every patient, upon their release, was provided a POPAE form, duly filled by the respective ward’s designated physician. Before this data secured a spot in our system, a senior attending would review and approve its contents. In instances where a patient was readmitted within a month of their primary surgery, the medical team was promptly notified. Cases presenting complexities were recurrently brought to the collective attention of the neurosurgical team during MMC meetings. This analysis prominently spotlighted octogenarians. We opted to exclude data from pediatric subjects and subjects younger than 80 years. Our definitions and criteria, integral to the methodology and interpretation of this study, have been previously delineated in depth in our earlier publications.^3,21^ This foundational work ensures consistency in the application and understanding of terms across our studies, allowing for comparability and continuity.

## Statistical Analysis

Categorical data were shown as counts and percentages. Continuous data, verified as normally distributed with the Shapiro–Wilk test, were depicted as means ± standard deviations. We utilized univariate analysis to contrast baseline and surgical attributes across groups. A binary logistic regression analysis model assessed potential risk determinants for AEs. We considered a *P*-value <.05 as statistically significant. All data processing was done with SPSS version 24.0.0.0 (IBM Corp., Armonk, NY, USA).

## Results

### Demographic Data and Baseline Characteristics

Between January 2020 and December 2022, a cohort of 184 patients, boasting an average age of 83.1 ± 2.8 years, underwent surgical procedures. A significant 81.5% (150/184) of these surgeries were elective in nature, while the remaining 18.5% (29/184) were categorized as emergency procedures. The primary driver for surgery in a majority of cases was degenerative pathologies, accounting for 69.0% (127/184) of the total. When assessed using the age-adjusted Charlson Comorbidity Index (CCI), the mean comorbidity score was 8.1 ± 2.2, with cardiac diseases emerging as the most prevalent ailment among the cohort. A comprehensive breakdown of the study’s population demographics can be found in Table 1.

**Table 1.** Baseline Characteristics.

	N = 184	%
Age, years (mean, SD,range)	83.1 (2.8; 80-96)	
Sex (n, %)		
Male	111	60.3
Female	73	39.7
Non-Elective	34	18.5
Elective	150	81.5
Spinal		
Degenerative	127	69.0
Tumor	15	8.2
Trauma	27	14.7
Infection	15	8.2
Comorbidities		
Age-adjusted CCI score (mean, SD)	8.1 (2.2)	
Arterial hypertension	96	52.2
Myocardial infarction	68	37.0
Coronary heart disease	75	40.8
Atrial fibrillation	55	29.9
Heart failure	40	21.7
Peripheral vascular disease	44	23.9
COPD	56	30.4
Diabetes mellitus Type II	57	31.0
Renal failure	54	29.3
Liver disease	28	15.2
Gastrointestinal ulcer	24	13.0
TIA/stroke	17	9.2
Malignancy	18	9.8
Dementia	28	15.2
Alcohol abuse	21	11.4
Localization		
Cervical	27	14.7
Cevicothoracic	2	1.1
Thoracic	16	8.7
Thoracolumbar	7	3.8
Lumbar	119	64.7
Lumbosacral	13	7.1

SD, standard deviation.

### Surgical Characteristics

As detailed in Table 2, a majority of patients, precisely 71.2%, underwent decompression procedures, with the remaining 28.8% receiving instrumentation surgeries. The dorsal approach emerged as the predominant surgical method, utilized in 83.7% of cases. On average, surgeries spanned a duration of 170.7 ± 92.9 minutes. The mean hospital stay post-surgery was 8.2 ± 4.3 days. Additionally, the collective rate of surgical revisions stood at 12.5%.

**Table 2.** Peri- and Postoperative Surgical Characteristics and Clinical Course Across 184 Patients Undergoing Surgery. Except Where Otherwise Indicated, Quantities are Mean (SD).

	n = 184
Surgical approaches (n,%)	
Decompression	131 (71.2)
Instrumentation	53 (28.8)
Ventral	10 (5.4)
Dorsal	154 (83.7)
Concorde	19 (10.3)
Dorsal and Side	1 (.5)
Intraoperative blood transfusion, n (%)	10 (18.4)
Surgical duration, min	170.7 (92.9)
No. of levels decompressed/fused	2.5 (.7)
Hospital stay, days	8.2 (4.3)
ICU Stay, days	.5 (2.3)
Mortality (n, %)	4 (2.2)

### Occurrence of Surgery-Related Aes

#### Degenerative Disease

The predominant AE in patients diagnosed with degenerative diseases was wound infection, accounting for 3.1% of cases. Importantly, every one of these infections necessitated revision surgery. Two patients suffered from postoperative bleeding, a revision surgery was performed in both cases. Remarkably, two patients exhibited new neurological deficits, and one experienced dural leakage that mandated a surgical revision. There were no fatalities recorded among these patients. Outside the realm of surgical-related AEs, urinary tract infections surfaced as the most common complication.

#### Tumor Disease

Wound infection and dural leaks presented with the same prevalence of 13.3%. Revision surgery was necessary in all cases with wound infection and dural leaks. The incidence of a second transfer to the ICU was 6.7 %, which was attributable to cardiac disease. One patient deceased due to an acute heart failure.

#### Trauma

Among patients diagnosed with spinal trauma, there was one instance each of wound infection and dural leak. Both complications required surgical revisions. It’s noteworthy that there were no instances of malpositioning of the implanted materials. Two patients, after being transferred to the ICU due to pulmonary embolism, did not survive. Each complication—acute renal failure, pneumonia, heart failure, and respiratory insufficiency—occurred in 7.4% of patients individually.”

#### Infected spine

Postoperative wound infection was the most prevalent AE in this group (26.7%), and 20.0% of affected patients required revision surgery. Dural leaks and postoperative infection were present in one case, respectively. One patient died caused by the development of sepsis.

A comprehensive breakdown of surgery-related and non-surgery-related AEs can be found in Tables 3 and 4, respectively. It’s noteworthy that the observed mortality rate of 2.2% was not attributed to surgical interventions.

**Table 3.** Summary of Surgery-Related Adverse Events.

	n	%	Revision Surgery	%
Wound event				
Degenerative	4	3.1	4	3.1
Tumor	2	13.3	1	6.7
Trauma	1	3.7	1	3.7
Infection	4	26.7	3	20.0
Dural leak				
Degenerative	1	0.8	1	0.8
Tumor	2	13.3	2	13.3
Trauma	1	3.7	1	3.7
Infection	1	6.7	1	6.7
Postoperative infection				
Degenerative	0	0.0	0	0.0
Tumor	1	6.7	0	0.0
Trauma	1	3.7	0	0.0
Infection	1	6.7	0	0.0
Malposition of implanted material				
Degenerative	1	0.8	1	0.8
Tumor	0	0.0	0	0.0
Trauma	0	0.0	0	0.0
Infection	0	0.0	0	0.0
New neurological deficits				
Degenerative	2	1.6	2	1.6
Tumor	0	0.0	0	0.0
Trauma	0	0.0	0	0.0
Infection	0	0.0	0	0.0
Rebleeding				
Degenerative	2	1.6	2	1.6
Tumor	0	0.0	0	0.0
Trauma	0	0.0	0	0.0
Infection	1	6.7	1	6.7
Surgical goal not achieved				
Degenerative	2	1.6	2	1.6
Tumor	0	0.0	0	0.0
Trauma	1	3.7	0	0.0
Infection	0	0.0	0	0.0
Others				
Degenerative	1	0.8	1	0.8
Tumor	0	0.0	0	0.0
Trauma	0	0.0	0	0.0
Infection	0	0.0	0	0.0
Secondary transfer to IMC or ICU				
Degenerative	0	0.0	0	0.0
Tumor	1	6.7	0	0.0
Trauma	2	7.4	0	0.0
Infection	1	6.7	0	0.0
Death				
Degenerative	0	0.0	----	----
Tumor	1	6.7	----	----
Trauma	2	7.4	----	----
Infection	1	6.7	----	----

IMC, intermediate care unit; ICU, intensive care unit.

**Table 4.** Non-surgery-related Adverse Events by Spinal Pathology.

	n	%
Degenerative (n, %)		
Acute renal failure	2	1.6
Respiratory deficiency	2	1.6
Heart failure	1	0.8
Pneumonia	2	1.6
Pulmonary embolism	1	0.8
Urinary tract infection	6	4.7
Tumor (n, %)		
Acute renal failure	0	0.0
Respiratory deficiency	0	0.0
Heart failure	1	6.7
Pneumonia	1	6.7
Pulmonary embolism	0	0.0
Urinary tract infection	0	0.0
Trauma (n, %)		
Acute renal failure	2	7.4
Respiratory deficiency	2	7.4
Heart failure	2	7.4
Pneumonia	2	7.4
Pulmonary embolism	2	7.4
Urinary tract infection	1	3.7
Infection (n, %)		
Acute renal failure	1	6.7
Respiratory deficiency	1	6.7
Heart failure	0	0.0
Pneumonia	2	13.3
Pulmonary embolism	0	0.0
Urinary tract infection	0	0.0

### Risk Factors for AEs

Binary logistic regression analysis revealed that the presence of comorbidities, longer operative times as well as instrumentation surgery were significant predictors for the occurrence of AEs mortality (Table 5).

**Table 5.** Risk Factors Associated With Surgery Related AEs.

Risk Factor	OR (95% CI)	*P*-value
Age-adjusted CCI score	1.8 (1.1-5.2)	**.002**
Duration of surgery	1.4 (1.1-1.9)	**.006**
Number of levels decompressed	.5 (.1-1.0)	.889
Surgical approach^a^	1.3 (1.1-2.1)	**.021**
Hospital stay	.5 (.1-1.5)	.778

CCI, Charlson Comorbidity Index; CI, confidence interval; OR, odds ratio. A *p*-value ≤ 0.05 inidcates statistically significant results.

^a^Instrumentation.

## Discussion

To our knowledge, this is the inaugural study offering a comprehensive account of AEs in octogenarians undergoing various spinal surgeries for diverse spinal pathologies, utilizing a large, prospectively accumulated database. Our data indicates that degenerative pathologies predominantly necessitate surgery in these patients, succeeded by traumatic incidents leading to spinal fractures. Notably, irrespective of spinal pathology, we found the overall incidence of surgery-related AEs to be 15.8%, with a subsequent 12.5% necessitating revision surgery. Concurrently, the overall rate of non-surgery related AEs stood at 16.3%. The prevalence of comorbidities, as gauged by the age-adjusted CCI, was alarmingly high at 8.1, underscoring a compromised preoperative clinical status, with cardiac ailments being the most common. Decompression surgery was the primary intervention for most patients. Interestingly, the aggregate mortality rate was 2.2%, which was not directly attributable to the surgical intervention. Elevated comorbidity rates, instrumentation surgery and longer operative times emerged as significant predictors of occurrence of AEs.^22-25^

Elevated comorbidity rates frequently indicate an augmented risk for postoperative AEs in elderly patients post-spinal surgery. A precedent study on patients aged 75 and above with degenerative lumbar pathologies found a correlation between pre-existing medical conditions and increased postoperative AEs, encompassing wound and systemic complications.^26^ Yet, in a subsequent study by the same research group, which assessed the safety profile of lumbar spine surgery due to degenerative pathologies in 26 patients over 85, it was posited that comorbidities weren’t the primary drivers of AEs. Instead, extended surgical durations were implicated.^27^ Such findings may be attributed to the rigorous patient selection for the study, which emphasized a satisfactory profile of underlying pre-existing conditions and excluded emergency surgeries. Our earlier research, which centered exclusively on octogenarians with diverse spinal pathologies—ranging from spinal epidural abscesses to tumorous afflictions—indicated that elevated comorbidity rates substantially amplify the risk of complications, and in some cases, mortality.^14-16,28,29^ Corroborating these findings, a retrospective analysis of 47 octogenarians with varied spinal pathologies identified comorbidity rates as a significant determinant for AEs, registering a prevalence of 17.0%. The 30-day postoperative mortality in this group was noted to be 2.1%, aligning with our current study’s findings. In the present investigation, we have furnished prospective data on 184 octogenarians post spinal surgery, reinforcing the pivotal role of comorbidity rates in influencing surgical outcomes. It is imperative to underscore the salience of developing a robust and consistent system for AE documentation, as practiced in our institution. Such a system can serve as a linchpin in averting AEs, priming physicians on potential pitfalls and equipping them with strategies to counteract complications. Therefore, instituting mechanisms to chronicle AEs prospectively and crafting standardized, evidence-based protocols is of paramount importance.

Our surgery-related morbidity rate stood at 15.8%, with wound complications being the most prevalent among all AEs. Post-surgical rebleeding also emerged as a significant adverse event, predominantly observed in patients on anticoagulants for atrial fibrillation. The association between anticoagulant use and postoperative re-bleeding after spinal surgery remains a topic of debate. For instance, in their study encompassing 3729 patients, Yi et al. inferred a significant link between postoperative hematoma and anticoagulation therapy.^30^ Echoing this, Lawton et al. highlighted, through an analysis of 30 patients, a correlation between postoperative spinal hematoma, prior spinal surgeries, and anticoagulation medication.^31^ Supporting these assertions, Kou et al. indicated that coagulopathy and advancing age increase the risk of postoperative spinal hematoma.^32^ It’s worth noting that, in our study, patients were administered antidotes in line with the German Guidelines [30] to counteract the anticoagulation effects. Yet, nearly 30% of our subjects exhibited renal failure. This suggests that compromised renal function may prolong the impact of anticoagulants, even after antidote administration. In addition, pneumonia and urinary tract infections dominated the non-surgical related AEs across diverse spinal pathologies, reinforcing the paramount importance of vigilant peri- and postoperative monitoring in this sensitive cohort to prevent potential life-threatening AEs. It is indispensable to maintain close collaboration with anesthesiologists in all cases. Moreover, when deliberating on surgical interventions aimed at enhancing the quality of life, one must factor in the patient’s life expectancy. Irrespective of age, individuals with longer anticipated life spans are better poised to reap the benefits of surgery and mitigate the repercussions of persistent spinal issues. Hence, there’s a pressing need for a standardized classification system, akin to the Clavien-Dindo Classification, as incorporated in our institutional protocol. Such a system could augment our data documentation and foster improved inter-center comparisons,^33-35^ facilitating timely therapeutic interventions and prevention of AEs.

Spinal instrumentation surgeries in octogenarians are inherently challenging due to the pronounced comorbidity profiles often found in this age group. Such patients frequently present with numerous health concerns, including cardiovascular diseases, diabetes, and pulmonary complications. It is well-documented that these comorbidities correlate with increased postoperative complications following spine surgeries. Our current study has several strengths that warrant emphasis.^36,37^ Foremost among these is the prospective nature of our data collection on each patient, which ensures the fidelity and comprehensiveness of the information we gathered. Specifically, our research highlighted that the occurrence of AEs was not merely associated with a higher prevalence of comorbidities. We found significant correlations between AEs and longer operative times, as well as with the type of surgery, specifically instrumentation surgery. This concurs with earlier studies of retrospective nature. For instance, Watanabe et al., in their prospective research on 270 octogenarians with lumbar degenerative diseases, similarly reported that the incidence of AEs was associated with prolonged operative times and instrumentation surgeries. Despite these challenges, they noted discernible benefits in clinical outcomes. Moreover, retrospective studies based on claims data in the elderly have echoed the findings of Watanabe et al.^38^ However, a glaring gap exists in the literature. The majority of retrospective studies are based on administrative data, focusing predominantly on degenerative diseases in the elderly. As such, AEs arising from more severe spinal conditions, such as tumors, trauma, or infections, remain underexplored. Consequently, strategies to mitigate these events are still in their infancy. In stark contrast, our study is pioneering, given that it is grounded on prospectively collected data. All patients details were meticulously discussed by the treating physicians. Such a rigorous approach augments the learning trajectory of medical residents, circumventing potential pitfalls of under-reporting or over-reporting of AEs. Additionally, complex cases were systematically presented and deliberated upon regular MMC meetings. We postulate that these rigorous methodologies contributed to our observed low morbidity and mortality rates, findings that resonate with those of Kashiwazaki et al., 2020.^39,40^ Through our methodical identification and documentation of AEs, we anticipate that the repetition of avoidable incidents will decrease. This approach established a robust foundation for crafting strategies and algorithms to further minimize such events in the future.

## Limitations

Our research has a distinct emphasis, focusing exclusively on octogenarians—a demographic that has been notably understudied in previous literature. This focus is particularly salient given the unique challenges posed by this age group, often characterized by diminished baseline physiological reserves, which can complicate surgical outcomes and post-operative recovery. The predominant strength of our study thus lies not only in its pioneering approach to documenting AEs via a prospectively established database covering a broad spectrum of spinal pathologies but also in shedding light on a segment of the population that has traditionally been overlooked. However, as with any research endeavor, certain limitations must be acknowledged. Primarily, our follow-up duration, capped at 30 days, restricts the range of complications we could observe, particularly lingering issues such as adjacent-level diseases following spinal fusion procedures. Secondly, despite rigorous oversight of each case, occasional interpretation errors are inevitable, potentially influencing the precision of event recordings. A third point of potential variability arises from our methodology. Incorporating a structured system like the SAVE-V2 classification might provide a more uniform platform, mitigating both inter- and intra-observer discrepancies. A further limitation of our study is its single-center design, which may limit the generalizability of our findings. Future research could benefit from a multicenter approach to enhance the applicability of the results across different populations and settings. The inherent heterogeneity of disease presentations and the variety of surgical procedures within our patient cohort, reflective of actual clinical practices, may introduce variability that could obscure the precision of the study’s outcomes. Moreover, the omission of preoperative and postoperative nutritional assessments represents a limitation, given the recognized influence of nutritional status on surgical recovery.

Lastly, while our study highlights the intricacies of treating octogenarians, the actual impact of educational interventions on the MMC meetings process remains intangible, presenting a challenge for an objective evaluation.

## Conclusions

Our study has determined that the incidence of AEs in spinal surgery within the octogenarian demographic stands at 15.8%. This incidence appears to be associated with factors such as increased comorbidity rates, longer durations of surgery, and the complex nature of instrumentation procedures that are often necessary in this age group. Notably, we found that the mortality rate was 2.2%, which our analysis suggests is not directly related to the surgical procedures but rather to prevalent underlying conditions. The results underscore the importance of meticulous record-keeping, as our findings reveal that systematic documentation is crucial in refining surgical approaches for elderly patients. By carefully documenting surgical outcomes, we can gain a deeper understanding of the risks and benefits associated with spinal surgeries in older populations.

## Data Availability

The datasets generated during and/or analyzed during the current study are available from the corresponding author on reasonable request.
